# Physician associates and GPs in primary care: a comparison

**DOI:** 10.3399/bjgp15X684877

**Published:** 2015-04-27

**Authors:** Vari M Drennan, Mary Halter, Louise Joly, Heather Gage, Robert L Grant, Jonathan Gabe, Sally Brearley, Wilfred Carneiro, Simon de Lusignan

**Affiliations:** Faculty of Health, Social Care and Education, Kingston University and St George’s University of London, London.; Faculty of Health, Social Care and Education, Kingston University and St George’s University of London, London.; Social Care Workforce Research Unit, King’s College London; Health Care Management and Policy, University of Surrey, Guildford.; Faculty of Health, Social Care and Education, Kingston University and St George’s University of London, London.; Centre for Criminology and Sociology, Royal Holloway, University of London, Egham.; Faculty of Health, Social Care and Education, Kingston University and St George’s University of London, London.; Directorate of Corporate Affairs, St George’s Healthcare NHS Trust, London.; Health Care Management and Policy, University of Surrey, Guildford.

**Keywords:** general practitioners, observational study, physician assistants, physicians, family, primary health care

## Abstract

**Background:**

Physician associates [PAs] (also known as physician assistants) are new to the NHS and there is little evidence concerning their contribution in general practice.

**Aim:**

This study aimed to compare outcomes and costs of same-day requested consultations by PAs with those of GPs.

**Design and setting:**

An observational study of 2086 patient records presenting at same-day appointments in 12 general practices in England.

**Method:**

PA consultations were compared with those of GPs. Primary outcome was re-consultation within 14 days for the same or linked problem. Secondary outcomes were processes of care.

**Results:**

There were no significant differences in the rates of re-consultation (rate ratio 1.24, 95% confidence interval [CI] = 0.86 to 1.79, *P* = 0.25). There were no differences in rates of diagnostic tests ordered (1.08, 95% CI = 0.89 to 1.30, *P* = 0.44), referrals (0.95, 95% CI = 0.63 to 1.43, *P* = 0.80), prescriptions issued (1.16, 95% CI = 0.87 to 1.53, *P* = 0.31), or patient satisfaction (1.00, 95% CI = 0.42 to 2.36, *P* = 0.99). Records of initial consultations of 79.2% (*n* = 145) of PAs and 48.3% (*n* = 99) of GPs were judged appropriate by independent GPs (*P*<0.001). The adjusted average PA consultation was 5.8 minutes longer than the GP consultation (95% CI = 2.46 to 7.1; *P*<0.001); cost per consultation was GBP £6.22, (US$ 10.15) lower (95% CI = −7.61 to −2.46, *P*<0.001).

**Conclusion:**

The processes and outcomes of PA and GP consultations for same-day appointment patients are similar at a lower consultation cost. PAs offer a potentially acceptable and efficient addition to the general practice workforce.

## INTRODUCTION

One solution to problems of medical staff shortages has been the development of ‘mid-level’ professionals, such as nurse practitioners and physician assistants.^1^ Although not doctors, these professionals have the education and training to diagnose, treat, and refer autonomously within practice boundaries, as specified by local legislation and/or their employing organisation.^2^ The physician assistant role has a 50-year history in the US, with growing numbers working in primary care^3^ and further expansion of training programmes under way in response to the Affordable Care Act.^4^ Modelled on the success of the role in the US, physician assistants have recently been introduced into other countries such as Canada, Australia, the Netherlands, Germany, and India.^5^ In the UK, the first physician assistants were trained in the US and new UK courses did not produce graduates until 2009.^6^ Unlike physician assistants in the US and the Netherlands, however, those in the UK do not have the legal authority to prescribe.^6^ Since 2013, the name of physician associates (PAs) has been adopted in preference to physician assistants in the UK. This change has been made to distinguish them in the UK setting where the name physician assistant has been given to healthcare assistants in hospital medical teams who are unqualified staff undertaking directed tasks, such as phlebotomy.

The NHS is primary care-led. GPs (family doctors) combine in small- and medium-sized businesses (practices) to provide general medical services to the local population. Most commonly, practices comprise four to six GPs, each with a list of some 1800 patients spanning all ages. GPs are the first point of contact for all health issues (other than emergencies) and refer patients to specialist secondary care. Few GPs employ PAs,^7^ and evidence on clinical outcomes or costs of PAs, from the US or elsewhere, is scant.^8^

This is a study of the use of PAs in general practices in England. As much of the work of PAs in this setting is attending to patients requesting a same-day or urgent appointment,^7^ the processes, outcomes, and costs of PAs fulfilling these functions were compared with those of GPs. This research was part of a larger study of PAs in primary care in England.^9^

## METHOD

### Setting and design

A comparative observational design was used, based on consultation records and a linked medical record review and patient satisfaction survey. The study was set in 12 volunteer general practices, six employing PAs and six not employing PAs, matched by practice size, sociodemographics of the practice population, and urban/rural geographical environment in the south, east, and southwest of England. Details of the staff in practices with and without PAs are given in Table 1.

**How this fits in**

Physician associates (previously known as physician assistants) are a new professional group in UK general practice, and evidence is required on their outcomes and costs. For patients attending for same-day or urgent appointments, PAs attended a younger patient group who present with less medically acute problems and fewer long-term conditions, compared to those attended by GPs. After adjusting for case-mix, there was no difference between PA and GP consultations in the rate of investigations, referral to secondary care, prescriptions issued, or the rate of patient re-consultation for the same or a closely related problem within 14 days. Patients report high levels of satisfaction with PA and GP consultations. The average PA consultation was longer than with a GP, although costs per consultation with a PA were lower.

**Table 1.** Staff resources (whole time equivalent) in practices with and without physician associates

**Variables**	**Practices employing PAs (*n* = 6)**		**Practices without PAs (*n* = 6)**	
	
	**Range**	**Mean**	**Range**	**Mean**
List size, number of patients, *n*	4316–15 000	9357	4385–13 635	9637

GPs, partners and salaried, *n*	3–9.3	5.3	2.4–7.1	4.9

Patients per GP, *n*	1233–2304	1818	719–4292	2339

Physician associates, *n*	1 (*n* = 5);		0	
	2 (*n* = 1)			

Nurse practitioners, *n*	0 (*n* = 3);		0 (*n* = 4);	
	1.4 (*n* = 1);		1 (*n* = 2)	
	1.0 (*n* = 2)			

Nurses, others in patient care (PN, HCA, phlebotomists)	1.2–6.5 (*n* = 6)	3.4 (median)	1.5–4.6	2.6

Management, other professional	1.35–2.75 (*n* = 6)	2.2 (median)	1.1–4.5	2.3

Secretarial, reception, clerical	4.6–11.6 (*n* = 6)	7.8 (median)	4.6–10.1	7.6

HCA = healthcare assistant. PA = physician associate. PN = practice nurse.

### Participants

All patients attending for same-day or urgent appointments with participating (volunteer) PAs or GPs (in non-PA practices) in designated sessions over 4 weeks (2 weeks in winter and 2 weeks in summer months) 2011–2012 were eligible for inclusion. Practice staff extracted the records of these patients from the practice electronic database, assigned a unique study identifier and anonymised the information before passing the data to the research team. Patients aged >16 years in these designated sessions were also offered a validated, patient satisfaction survey for general practice^10^ with their study ID by the practice staff and returned by post to the research team.

All but one of the PA-employing practices had some guidelines for the receptionists to assign patients, who were requesting same-day appointments, to PAs. These guidelines were defined by the supervising GP and were based on the knowledge and experience of the individual PA. They ranged from all patients, except those aged <1 year, to a reception-held list of patient problems indicating which were suitable for a GP, PA, or nurse practitioner appointment, dependent on the staff available for that surgery. In five of the practices, PAs were given either longer appointment time slots than GPs (although shorter than the nurses) or the same time slots plus empty appointments to ensure enough time to consult their supervising GP if required.

### Data and outcome measures

The primary outcome was patient re-consultation within 14 days for the same or a linked problem, which could indicate any difference in consequences for the patient and also the practice and doctor workload. This outcome has been used previously in a UK study of nurse practitioner consultations for same-day appointments compared with GPs.^11^ Secondary outcome measures were processes of care (diagnostic tests ordered, referrals made, prescriptions issued, general advice, and medication management advice given); patient satisfaction; and length and cost of consultation. Other variables that were plausible confounders were age, medical acuity of presenting problem, sex, number of times attending the practice in the previous 3 months, number of problems, number of chronic disease registers, and socioeconomic deprivation.

Data collection for these outcome measures and confounders covered:

- background information about each patient (date of birth, sex, ethnic group, post [zip] code [from which a deprivation index can be obtained],^12^ comorbidities (from the pay-for-performance registers used in the NHS), repeat prescriptions, frequency of contact with the practice over the previous 3 months);
- information regarding the same-day/urgent consultation of interest and any re-consultation for the same or a linked problem at the practice or an urgent care facility within 14 days of index consultation (the presenting problem/s; processes of care [tests, referrals, general advice, and medication management advice given, and prescriptions issued] and length of consultation in minutes [time in and time out on electronic record]; and free text comments made by the clinician); and
- Self-reported patient satisfaction.

All except patient satisfaction were collected from routinely held practice patient records. To examine the case mix by medical acuity and complexity of the consulting patients, a classification^13^ was assigned to each presenting problem:

- acute (that is, medically defined as something with a rapid onset sometimes representing severe disease);
- chronic;
- minor problem or symptoms;
- prevention (for example, malaria protection advice for travel); or
- process of care (for example, provision of a medical certificate).

Records with a re-consultation for the same or linked problem within 14 days formed a sub-sample for a medical record review. The medical record review addressed the question as to whether, on the basis of the written record and in the light of the subsequent consultation(s), the index consultation was judged as appropriate by an experienced GP. One GP in the team led the review with four independent experienced GPs, using the framework of Weed’s problem-orientated medical record.^14^ The GP reviewers were blinded as to whether the record was of a GP or PA consultation. One-tenth of records were reviewed by two GPs to test for reliability.

### Sample size

The sample size was based on randomised controlled trial data which compared the rate of re-consultation within 14 days for same-day consultations (the study’s main outcome) with those of a nurse practitioner and GP.^11^ A sample size of 205 consultations with a PA and 205 with a GP was required to give 80% power at a significance level of 5% for a multilevel logistic regression, adjusting for age and sex. To analyse the main outcome by patient satisfaction, the number of participants required was based on an anticipated minimum 30% response rate to the patient survey, resulting in an estimation of approximately 600 individual patients required.

### Analysis

Generalised estimating equation models^15^ were used to assess differences in processes and outcomes of consultations and patient satisfaction between PAs and GPs, adjusting for variables that were significant predictors or notably confounded the relationship with the dependent variable or were markedly different in the PA patients and the GP patients; for example, age. These multilevel models account for clustering that indicates inter-practice differences. Poisson models were fitted to counts, binomial to dichotomous outcomes, and proportional odds (checked graphically by plotting the cumulative logarithm of the odds ratio) for patient satisfaction, which was ordinal. All ratios compare PAs against GPs as the baseline, hence a ratio of two would indicate that PAs were twice as likely as GPs to achieve a process or outcome. An overall difference in consultation length between the professions, averaged over the distribution of age and the presenting problem classification, was calculated using marginal effects.^16^ Nationally validated unit costs, 2011,^17^ were applied to these adjusted consultation lengths to calculate consultation costs. PAs were costed as Band 7 nurses, following advice from the UK Association of Physician Associates (personal communication, J Gray, 2012). Other ways in which the use of PAs may affect practice costs were also considered, such as GP time spent in supervision and training, but could not be quantified as data were not available. Analysis was carried out using SPSS software (version 21) and Stata software, (version 11.2).

## RESULTS

Anonymised clinical records of 2086 patients attending for same-day appointments in the designated sessions in the 12 practices were given to the research team. Of these, 932 (44.7%) had the index consultation with a PA and 1154 (55.3%) with a GP (Table 2).

**Table 2.** Characteristics of patients consulting PAs and GPs

	**Patients consulting PAs**		**Patients consulting GPs**		***P*-value**
	
	**Patients with data (*n* = 932), *n***	**Mean (SD)**	**Patients with data (*n* = 1154), *n***	**Mean (SD)**	
Age, years	929	34.35 (23.20)	1154	42.93 (24.87)	<0.001

Index of Multiple Deprivation	929	21.99 (16.61)	1148	15.81 (13.04)	<0.001

Female	930	548 (58.9%)	1154	686 (59.4%)	0.81

White British ethnic group	638	420 (65.8%)	746	528 (70.8%)	0.05

Number of chronic disease registers per patient	932	0.55 (0.99)	1152	0.87 (1.26)	<0.001

Number of repeat prescriptions	931	1.81 (3.02)	1154	2.60 (3.63)	<0.001

Number of visits to practice in previous 3 months	931	2.12 (2.83)	1154	2.70 (2.99)	<0.001

**Presenting problem classification**	**Patients with data, *n***	**Category, *n* (%)**	**Patients with data, *n***	**Category, *n* (%)**	

Acute	932	34 (3.6)	1154	61 (5.3)	<0.001
Chronic	932	305 (32.7)	1154	504 (43.7)	(for all categories)
Minor/symptoms	932	586 (62.9)	1154	579 (50.2)	
Prevention	932	1 (0.1)	1154	3 (0.3)	
Process	932	6 (0.6)	1154	7 (0.6)	

PA = physician associate.

### Study population

Patients seeing a PA were significantly younger, lived in more deprived neighbourhoods, were on fewer chronic disease registers, received fewer prescriptions, and made fewer visits to the practice over the preceding 3 months (Table 2). PAs attended significantly more patients presenting for ‘minor problems or symptoms’ and less often ‘chronic’ problems than GPs (Table 3).

**Table 3.** Process and outcome measures for PA consultations compared with GP consultations

	**Unadjusted**		**Adjusted**		***P*-value**
	
	**Rate ratio**	**95% CI**	**Rate ratio**	**95% CI**	
Re-consultation for the same and linked problem^a^	1.25	0.89 to 1.77	1.240	0.86 to 1.79	0.247
Diagnostic tests ordered^b^	1.06	0.82 to 1.37	1.08	0.89 to 1.30	0.44
Referrals^c^	0.84	0.57 to 1.22	0.95	0.63 to 1.43	0.80
Prescriptions issued^d^	1.18	0.86 to 1.60	1.16	0.87 to 1.53	0.31

	**Odds ratio**	**95% CI**	**Odds ratio**	**95% CI**	***P*-value**

General advice given^e^	3.56	2.95 to 4.30	3.30	1.69 to 6.45	<0.001
Advice on medication management^e^	1.43	1.12 to 1.82	1.72	1.08 to 2.73	<0.022

	**Mean difference**	**95% CI**	**Mean difference**	**95% CI**	***P*-value**

Consultation duration, minutes^f^	5.5	4.8 to 6.2	5.8	2.5 to 7.1	<0.001
Cost per consultation, GBP 2011	N/A	N/A	−6.22 (US$ 10.15)	−7.61 to −2.46	<0.001

Adjustment made for clustering at practice level (all analyses).

a Presenting problem classification, age, number of times at practice in previous 3 months.

b Presenting problem classification, age, number of problems, number of chronic disease registers, sex, deprivation.

c Practice type, PA study condition classification, age;

d Practice type, PA study condition classification, age, number of problems.

e Presenting problem classification, age, number of times at practice in previous 3 months, number of presenting problems.

f Presenting problem classification, age, number of presenting problems. N/A = not applicable.

### Re-consultation rates: primary outcome

A total of 514 patients (24.6%) re-consulted within 14 days for the same or linked problem to the index consultation (the primary outcome). There was no significant difference in re-consultation rates between those who initially consulted PAs or GPs (Table 3). This result was also non-significant when adjusting for whether a re-consultation was planned (rate ratio 1.05, 95% confidence interval [CI] = 0.75 to 1.48, *P* = 0.76). Among 932 index consultations with a PA, 229 (24.6%) had a planned re-consultation, while this was the case for 214 out of 1153 (18.6%) index consultations with a GP.

### Consultation processes: secondary outcomes

Once adjusted for clustering at practice level, patient age, presenting problem classification, and other covariates of relevance, there was no significant difference between PAs and GPs in the rate of diagnostic tests ordered, referrals to secondary care, or prescriptions issued, while PAs were significantly more likely to document giving general advice and advice on medication management to the patient (Table 3).

### Length of consultation and costs

The length of consultation was available for 1812 (86.9%) consultations (PA, *n* = 896 (49.4%), missing *n* = 36; GP, *n* = 916 (50.5%), missing *n* = 238). The crude duration times of PA consultations were mean 16.8 minutes, SD 8.3, median 15, IQR 11–21, and range 1–60. Among GPs, the corresponding statistics were mean 11.3 minutes, SD 7.6, median 10, IQR 7–14, and range 1–97. Marginal effects, averaging over the patient characteristics, yielded consultation times of 17.03 minutes for PAs and 11.23 for GPs (Table 2). GPs saw about three patients for every two seen by PAs, but GP salary and related costs were higher (GBP 3.08 [US$ 5.00] versus GBP 1.67 [US$ 2.73] per minute^14^). Hence, costs per consultation with PAs were lower than those with GPs (GBP 28.14 [US$ 45.92] versus GBP 34.36 [US$ 56.07]), a difference of GBP 6.22 (US$ 10.15) (Table 3).

### Patient satisfaction survey

Of 1020 patients aged >16 years, 539 (52.8%) returned a patient satisfaction survey: 220 (40.8%) had consulted a PA; 319 (59.2%) a GP. There were high rates of reported satisfaction (Table 4) with no significant difference between PA and GP consultations (Table 4). Most of those consulting a PA responded that they would be willing to consult a PA again (87.3%, 192/220), while 4.1% (9/220) definitely preferred to consult a GP, and the remainder did not express a preference.

**Table 4.** Reponses in patient satisfaction survey

**General satisfaction with the care**	**Response option**						**Crude (Mann-Whitney) *P*-value**	**Adjusted odds ratio**	**95% CI**	***P*-value**
	**Very satisfied, % (*n*)**	**Satisfied, % (*n*)**	**Neither satisfied nor dissatisfied, % (*n*)**	**Dissatisfied, % (*n*)**	**Very dissatisfied, % (*n*)**	**Missing, *n***				
PA	75.9 (164)	18.5 (40)	5.1 (11)	0	0	5	0.82	1.00	0.42 to 2.36	0.99
GP	76.5 (241)	19.4 (61)	3.2 (10)	0.6 (2)	0.3 (1)	4				

**Judgement of practitioner for:**	**Very good, % (*n*)**	**Good, % (*n*)**	**Neither good nor poor, % (*n*)**	**Poor, % (*n*)**	**Very poor, % (*n*)**	**Doesn’t apply, % (*n*)**	**Crude (Mann-Whitney) *P*-value**	**Adjusted odds ratio**	**95% CI**	***P*-value**

**Giving time**										
PA	74.5 (161)	22.7 (49)	2.8(6)	0	0	0	0.97	0.97	0.48 to 1.94	0.93
GP	74.4 (232)	21.8 (68)	2.6 (8)	1.0 (3)	0	0.3 (1)				

**Asking about symptoms**										
PA	71.5 (153)	24.3 (52)	3.7 (8)	0	0.5 (1)	0	0.39	1.19	0.62 to 2.29	0.60
GP	74.0 (231)	19.6 (61)	4.2 (13)	0.3 (1)	0.3 (1)	1.6 (5)				

**Listening**										
PA	73.0 (157)	22.8 (49)	4.2 (9)	0	0	0	0.20	1.30	0.63 to 2.68	0.47
GP	77.3 (242)	19.2 (60)	2.2 (7)	0.6 (2)	0	0.6 (2)				

**Explaining tests and treatments**										
PA	63.1 (135)	22.4 (48)	6.5 (14)	0.5 (1)	0	7.5 (16)	0.63	1.07	0.84 to 2.08	0.84
GP	64.2 (197)	21.5 (66)	4.9 (15)	0.7 (2)	0.3 (1)	8.5 (26)				

**Taking patient seriously**										
PA	72.6 (156)	21.4 (46)	5.1 (11)	0	0.5 (1)	0.5 (1)	0.57	1.12	0.66 to 1.93	0.67
GP	73.4 (229)	18.6 (58)	4.2 (13)	1.0 (3)	0.3 (1)	2.6 (8)				

**Involve in decisions**										
PA	56.1 (120)	24.8 (53)	5.6 (12)	1.9 (4)	0	11.7 (25)	0.53	1.10	0.73 to 1.67	0.65
GP	59.7 (185)	20.6 (64)	737 (24)	0.6 (2)	0.3 (1)	11.0 (34)				

**Treating with care**										
PA	72.9 (156)	22.4 (48)	4.7 (10)	0	0	0	0.67	1.09	0.59 to 2.01	0.79
GP	72.9 (226)	20.6 (64)	3.2 (10)	1.0 (3)	0	2.3 (7)				

### Clinical review of records of re-consulting patients for the same or linked problem

A clinical review was completed for 475 (93.0%) of 511 re-consulting patients: the others being used for training or assessing inter-rater reliability (found to be very good or good by review component [range k = 0.69 to k = 1.00]).

In the light of the re-consultation, records of the index consultation were judged to be appropriate for significantly more of the PA consultations than GPs (81.6% versus 50.8%), (Table 5). The GP reviewers incorrectly judged 58.3% (123/211, missing 12) of the PA consultations to be those of GPs and 23% (57/245, missing 7) of GP consultations to be those of PAs.

**Table 5.** Clinical records (including WEED’s elements^14^ therein) assessed as appropriate by independent GP reviewers

	**PA records (*n* = 223) *n*/*N* (%)**	**GP records (*n* = 252) *n*/*N* (%)**	***P*-value**
Overall appropriateness of the consultation record	182/223 (81.6)	128/252 (50.8)	<0.001

**Elements within the consultation record**			
Subjective information	174/223 (78.0)	94/251 (37.5)	<0.001
Objective information	168/223 (75.3)	93/252 (36.9)	<0.001
Assessment/problem	184/223 (82.5)	150/252 (59.5)	<0.001
Plan: investigation	187/223 (83.9)	145/252 (57.5)	<0.001
Plan: prescription	164/223 (73.5)	126/252 (50.0)	<0.001

## DISCUSSION

### Summary

This is the first major study of the work of PAs in primary care.^8^ No significant differences were found in rates of re-consultation or process measures (diagnostic tests, referrals, or prescriptions) between patients who had consulted a PA or GP for a same-day/urgent appointment, when adjusted for confounders and practice-level clustering. The PAs in this study attended younger and medically less complex patients than the GPs. High levels of patient satisfaction with PA consultations were found. A higher percentage of PA records of re-consulting patients were judged appropriate than GP records by independent GPs successfully blinded to the professional. In the view of the GP reviewers at debriefing after being unblinded, PAs made more thorough consultation records. The reviewers speculated whether this reflected PA training or the difference in length of appointments. They noted that they had not identified any unsafe practice. On average, the consultation times with PAs were longer than with GPs. This difference reflects appointment times set in the study practices, which were 10 minutes for GPs and 15 minutes for PAs (20 minutes in one practice), and may account for why PAs were able to provide fuller records and document significantly more advice-giving than GPs. As salary and related costs of GPs are higher than those of PAs, the cost of a GP consultation exceeded that of a PA by some GBP £6.22 (US$ 10.15). In adjusted analyses that controlled for variations in the case mix of GPs and PAs, no significant differences were found between the professionals in rates of re-consultation, referral to secondary care, prescribing, ordering investigations, or undertaking procedures, so no attempt was made to cost these activities.

### Strengths and limitations

This study is an observational study rather than a randomised controlled trial, and this may be seen as a weakness. The design was chosen to capture the impact of a new type of professional within the realities of general practice provision in the NHS, however, and multiple variables were collected to enable confounding to be adjusted for in all analyses. The PAs were volunteer participants in the research and that may been seen as a limitation, although it is not unusual in studies that compare consultations by different groups of professionals. Use of clinical records reduced the data collection burden and minimised the extent of missing data, but may be viewed as less robust than a prospective study. All records in the designated sessions were used rather than just those with surveys, as per the original protocol, as there was loss of fidelity to protocol in offering surveys in some practices at busy periods. The use of all records precluded any unintentional biases. The economic analysis was limited to considering consultation times, and the total cost of treatment was not calculated. Rates of referrals to secondary care, prescriptions, and re-consultations were not significantly different between professionals, however, so this may not have affected the overall conclusions. Lack of data on time spent by the designated GP on supervising and professional development of PAs, and signing of PA prescriptions, means that the true costs of PAs are underestimated to an unknown extent.

The medical record review and patient condition severity classification system used in this study both built on established methods but were novel for this study and require further validation.

### Comparison with existing literature

The PAs in this study attended a different patient case mix from the GPs and this is consistent with reports on the work of PAs from the US^18^,^19^ and the Netherlands.^20^ The reported high levels of patient satisfaction accords with reports in a survey of Medicare recipients in the US.^21^

The patient records made by GPs were less detailed than those of PAs. Other studies have identified problems in the quality and completeness of general practice records^22^,^23^ and consequent medicolegal issues.^24^

### Implications for research and practice

This study offers evidence to clinicians, managers, and commissioners of primary care services in the NHS as to the acceptability, effectiveness, safety, and costs of PAs when substituting for doctors for part of the primary care workload. Primary care is a key element of many healthcare systems facing changing demography, increased populations with chronic diseases, and financial challenges.^25^ There are growing concerns as to the availability of doctors to work in primary care internationally,^26^–^28^ and PAs may offer one potential solution, with their shorter duration in training compared with GPs and attendant lower salaries, as part of skill-mixed primary care teams. Within the UK, issues such as legal authority to prescribe need attention if the potential for using PAs in primary care is to be fully realised.

The findings of this study suggest that PA consultations, for same-day appointment patients, in general practices in England, result in similar outcomes and processes for similar consultations by GPs at a lower consultation cost. Deployment of PAs to attend patients, aligned with their competencies, could free up GP time to concentrate on more complex cases. PAs have the potential to be an asset to the primary care workforce in healthcare systems looking to strengthen their primary healthcare provision in the face of shortages of doctors, increasing demands, and financial stringency.
